# Fetal growth in PCOS pregnancies: dynamic evidence of restriction and the modifying role of ART

**DOI:** 10.3389/fmed.2026.1857905

**Published:** 2026-07-15

**Authors:** Heze Xu, Yijia Liu, Xinyi Bian, Zhijiao Wang, Zhen Li, Hang Zhen, Shanwei Xing, Jingshuang Zhou, Zekai Bai, Jiapo Li, Chong Qiao

**Affiliations:** 1 Department of Obstetrics and Gynecology, Shengjing Hospital, China Medical University, Shenyang, China; 2 Key Laboratory of Maternal-Fetal Medicine of Liaoning Province, Shenyang, Liaoning, China; 3 Research Center of China Medical University Birth Cohort, Shenyang, Liaoning, China; 4 Qingdao Women and Children's Hospital, Qingdao University, Qingdao, Shandong, China

**Keywords:** assisted reproductive technology, cohort study, fetal growth restriction, fetal growth trajectory, polycystic ovary syndrome

## Abstract

**Background:**

Polycystic ovary syndrome (PCOS), the most prevalent endocrine disorder among women of reproductive age, is associated with adverse pregnancy outcomes. However, the dynamic trajectory of fetal growth in PCOS pregnancies remains inadequately characterized.

**Objective:**

This study aimed to longitudinally assess fetal growth in PCOS pregnancies and evaluate whether assisted reproductive technology (ART) modifies associated fetal growth restriction.

**Methods:**

In this prospective multicenter cohort study (UNIHOPE; NCT03220750), 12,189 advanced maternal age pregnancies (364 with Rotterdam-diagnosed PCOS) underwent longitudinal fetal biometry in the second and third trimesters following ISUOG guidelines. Z-scores were derived using the study’s advanced-age reference, adjusting for gestational age and sex. Generalized linear models were adjusted for maternal demographics, lifestyle factors, and gestational diabetes mellitus, with subgroup analyses stratified by ART conception.

**Results:**

Among 12,189 advanced maternal age pregnancies (364 PCOS), PCOS fetuses showed reduced second-trimester Z-scores for femur length (MD: −0.13; 95% CI, −0.25 to −0.02) and head circumference (MD: −0.14; 95% CI, −0.23 to −0.04), with third-trimester deficits extending to biparietal diameter (MD: −0.18) and abdominal circumference (MD: −0.16). Birth length (MD: −0.16) and weight (MD: −0.13) Z-scores were lower in PCOS neonates. Growth restriction was evident only in spontaneous conceptions, with no significant differences in ART-conceived pregnancies. Findings were robust to GDM adjustment.

**Discussion:**

Fetal growth restriction in PCOS pregnancies is detectable from the second trimester and persists to delivery. ART conception appears to mitigate this restriction, yielding fetal growth parameters comparable to the reference population. These findings underscore the necessity of dynamic ultrasound surveillance in PCOS pregnancies and highlight a potential protective role of ART in perinatal outcomes.

**Clinical trial registration:**

https://clinicaltrials.gov, identifier NCT03220750.

## 1 Introduction

Polycystic ovary syndrome (PCOS) is the most prevalent endocrine disorder among women of reproductive age, affecting the physical and mental health of approximately 11–13% of the global female population (1). The condition is characterized by ovulatory dysfunction, hyperandrogenism, and polycystic ovarian morphology (2) and is frequently accompanied by insulin resistance (3), infertility (4), obesity (5), depression (6), and anxiety (7). Women diagnosed with PCOS frequently exhibit diminished fertility, a consequence of ovulatory dysfunction (8). Furthermore, PCOS-specific endocrine-metabolic disturbances, including hyperandrogenism (8), insulin resistance (3), and chronic inflammation (9), may exacerbate pregnancy risks through multiple mechanisms. These multifaceted pathological alterations not only significantly impair female fertility but also exert complex and long-term effects on pregnant individuals and their offspring (10), including gestational diabetes mellitus (11), fetal growth restriction (12), and preterm birth (12). Consequently, PCOS poses a significant challenge in the domain of perinatal medicine.

Previous research has focused on the impact of PCOS pregnancies on fetal development. Several studies indicate that neonates born to mothers with PCOS have an elevated likelihood of preterm birth, fetal growth restriction (12), and low birth weight (2, 13). Conversely, other reports suggest an absence of association between PCOS and fetal growth restriction (13, 14). Nevertheless, it is undeniable that these studies consistently acknowledge a correlation between PCOS and lower mean birth weight as well as fetal growth restriction.

However, the majority of extant research has concentrated on late-pregnancy outcomes in PCOS, with a paucity of dynamic monitoring of intrauterine fetal growth trajectories. This monitoring gap carries significant clinical implications, including the potential influence of metabolic abnormalities in PCOS patients, such as insulin resistance and hyperandrogenism, on placental development and fetal growth patterns as early as the first trimester. The utilization of dynamic monitoring has the potential to facilitate the earlier detection of deviations in fetal growth, thereby enabling timely intervention. This, in turn, could optimize pregnancy management and perinatal outcomes for women diagnosed with PCOS. The objective of this study is to provide insights into the intrauterine fetal development in PCOS patients.

Moreover, previous research has predominantly concentrated on PCOS populations undergoing assisted reproductive technology (ART), while attention to naturally conceived PCOS patients remains comparatively limited. The present study aims to address this research gap by investigating the effects of different conception modes on pregnancy and fetal outcomes in individuals with PCOS.

The present study focuses on PCOS patients, integrating ultrasound scans during pregnancy and comparing PCOS patients’ newborns with a control group’s newborns’ anthropometric measurements. The aim is to explore the relationship between PCOS and the growth restriction of fetuses during the relevant period. Furthermore, the study compares the anthropometric measurements of fetuses from PCOS patients treated with ART and those from naturally conceived PCOS patients. It also analyses the growth and development of fetuses after treatment with ART. Firstly, the study emphasises the importance of monitoring fetal growth during pregnancy and optimizes the management of PCOS patients during pregnancy to improve fetal health. Secondly, it provides strong evidence in support of ART treatment for PCOS patients and lays the foundation for future research on the long-term effects of ART on PCOS patients’ offspring.

## 2 Materials and methods

### 2.1 Study design and participants

**Figure 1:**
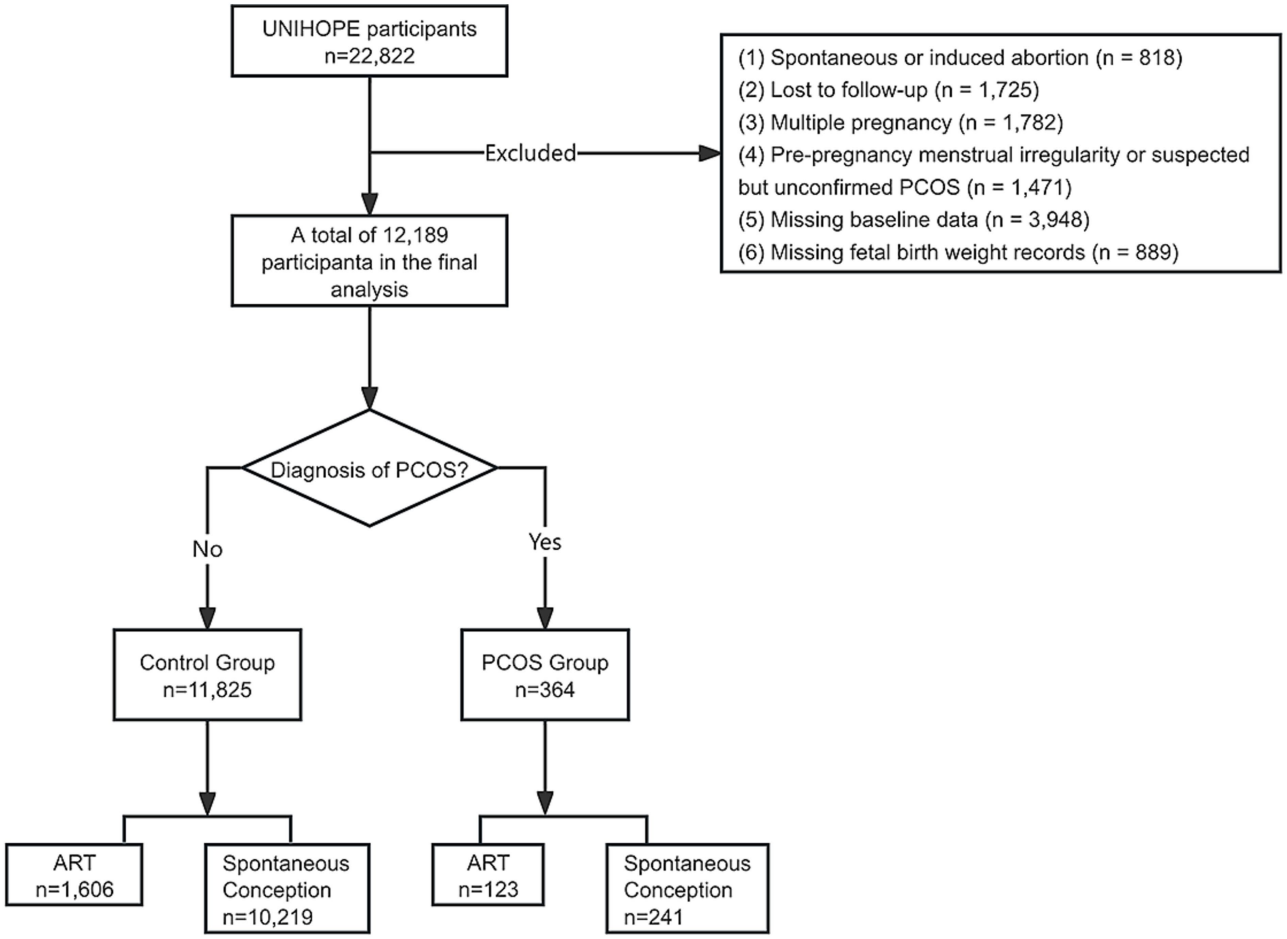
Flowchart of participant selection and study groups from the UNIHOPE cohort. This diagram illustrates the participant selection process for the present analysis within the prospective UNIHOPE (University Hospital Advanced Age Pregnant) cohort (ClinicalTrials.gov: NCT03220750). From an initial enrollment of 22,822 pregnant women, participants were excluded based on the following criteria: (1) spontaneous or induced abortion (*n* = 818), (2) lost to follow-up (*n* = 1,725), (3) multiple pregnancy (*n* = 1,782), (4) pre-pregnancy menstrual irregularity or suspected but unconfirmed PCOS (*n* = 1,471), (5) missing baseline data (*n* = 3,948), and (6) missing fetal birth weight records (*n* = 889). A total of 12,189 participants were included in the final analysis. These individuals were stratified based on a confirmed diagnosis of Polycystic Ovary Syndrome (PCOS) according to the Rotterdam criteria, forming a Control Group (n = 11,825) and a PCOS Group (*n* = 364). Each group was further subdivided according to conception mode: spontaneous conception or conception via Assisted Reproductive Technology (ART). The resulting four subgroups were: Control-ART (*n* = 1,606), Control-Spontaneous (*n* = 10,219), PCOS-ART (*n* = 123), and PCOS-Spontaneous (*n* = 241).

As shown in Figure 1, the study population was derived from the UNIHOPE (University Hospital Advanced Age Pregnant) cohort, a prospective, multicenter cohort study (15) (ClinicalTrials.gov identifier: NCT03220750). The parent UNIHOPE study enrolled 22,822 pregnant women between June 2017 and December 2020 across nine obstetric centers in seven major Chinese cities (Beijing, Shanghai, Guangzhou, Shenyang, Wuhan, Chongqing, and Chengdu). For this analysis, the following exclusion criteria were applied: natural or induced abortion (*n* = 818), Loss to follow-up (*n* = 1,725), multiple gestation (*n* = 1,782), pre-pregnancy menstrual irregularities or suspected but unconfirmed PCOS (*n* = 1,471), missing baseline patient information (*n* = 3,948), and missing fetal birth weight data (*n* = 889). After these exclusions, 12,189 pregnant women were included in the final analysis.

The primary exposure was the presence of pre-pregnancy PCOS. Diagnosis was based on the Rotterdam criteria (2). Trained personnel performed initial screening according to the following three criteria: Oligo- or anovulation, defined as amenorrhea (absence of menstrual bleeding for >182 days) or oligomenorrhea (menstrual cycle length >35 days or <8 cycles per year). Polycystic ovarian morphology, defined as the presence of ≥12 follicles (diameter 2–9 mm) in one or both ovaries and/or an increased ovarian volume (>10 cm^3^), assessed by pre-pregnancy ultrasound. Hyperandrogenism: Clinical hyperandrogenism was defined as a modified Ferriman-Gallwey score ≥5. Biochemical hyperandrogenism was defined as a free androgen index >4.5 and/or a total testosterone level >3.0 nmol/L. Participants who underwent standardized screening and met the pre-defined criteria were subsequently diagnosed with PCOS. Participants with ovulatory dysfunction who did not undergo or refused the standardized screening were classified as having suspected PCOS and were excluded from this study (*n* = 1,471). Among the 12,189 pregnant women of advanced age, 364 (3.0%) were diagnosed with PCOS.

### 2.2 Outcomes

This was a multicenter, prospective cohort study. Data were collected from nine clinical centers with extensive experience in obstetric ultrasonography. All ultrasound examinations were performed by physicians from each center who had over 5 years of experience in obstetric ultrasound and had received unified training prior to the study. Examinations were conducted in the second and third trimesters, following the recommendations of the International Society of Ultrasound in Obstetrics and Gynecology (ISUOG) guidelines (16, 17). Standard fetal biometric parameters were measured, including biparietal diameter (BPD), head circumference (HC), abdominal circumference (AC), and femur length (FL). All measurements strictly adhered to internationally recognized standard planes and protocols: BPD and HC: Measured in a transverse section of the fetal head at the level of the thalami, third ventricle, and cavum septum pellucidum. BPD was measured from the outer edge of the proximal parietal bone to the inner edge of the distal parietal bone. AC: Measured in a transverse section of the abdomen at the level of the stomach bubble and the intrahepatic portion of the umbilical vein, ensuring a complete abdominal contour with a clear outer skin edge. FL: Measured with the ultrasound beam perpendicular to the long axis of the femur, obtaining the maximum distance between the two ossification centers (18). After delivery, neonatal weight and length were recorded from the medical records. Given the potential for different fetal growth patterns in pregnancies of advanced maternal age, z-scores for all ultrasound biometric parameters, birth weight, and birth length were calculated based on the study’s advanced-age population, accounting for gestational age and fetal sex.

#### 2.2.1 Large for gestational age (LGA)

Defined as a birth weight >90th percentile for gestational age according to Chinese reference data. *Small for Gestational Age (SGA)*: Defined as a birth weight <10th percentile for gestational age. *Fetal Growth Restriction (FGR)*: Defined as a fetal abdominal circumference below the 10th percentile for gestational age. Low birth weight (LBW): Defined as a birth weight of less than 2,500 g. FGR is a prenatal sonographic diagnosis reflecting pathological growth deviation, whereas SGA and LBW are both postnatal classifications based on birth weight—SGA defined by percentiles and LBW by an absolute weight cutoff (19).

### 2.3 Covariates

The following potential confounders were adjusted for in the regression analysis: maternal age, pre-pregnancy body mass index (BMI), smoking status, alcohol consumption, exposure to secondhand smoke, parity, annual household income, educational level, and gestational diabetes mellitus (GDM). Height and self-reported pre-pregnancy weight were used to calculate BMI. Data on smoking, alcohol use, and secondhand smoke exposure were self-reported. Information on parity and educational level was obtained from medical records. ART refers to medical techniques involving the *in vitro* handling of human gametes (oocytes/sperm) or embryos to achieve pregnancy (WHO, 2022), primarily including in vitro fertilization and embryo transfer (IVF-ET), intracytoplasmic sperm injection (ICSI), and frozen–thawed embryo transfer (FET) (20).

### 2.4 Statistical analysis

Statistical analyses were performed using R software (version 4.4.1). Maternal baseline characteristics were compared using independent two-sample *t*-tests for continuous variables (presented as mean ± standard deviation) and Pearson’s chi-square tests for categorical variables (presented as frequency). To analyze the outcomes, generalized linear models (GLMs) were used to compare neonates from the PCOS group with those from the reference group. Two models were constructed:

#### 2.4.1 Model 1 and Model 2

Adjusted for maternal age, pre-pregnancy BMI, smoking, alcohol consumption, secondhand smoke exposure, parity, annual income, and educational level. Model 2: Further adjusted for GDM in addition to all covariates in Model 1.

Since the calculation of z-scores already accounted for fetal sex and gestational age, these variables were not included in the models. Due to the impact of PCOS on fertility, a subgroup analysis stratified by the use of ART was performed. Results are presented as mean differences (MD) with 95% confidence intervals (CIs). A two-sided *p*-value < 0.05 was considered statistically significant.

## 3 Results

As shown in Table 1, this study included a total of 12,189 pregnant women of advanced age, comprising 11,825 in the control group and 364 in the PCOS group. Regarding the use of ART, 1,606 women in the control group and 123 in the PCOS group conceived through ART. Our data showed that the mean pre-pregnancy BMI of mothers in the PCOS group was 23.68 kg/m^2^ (SD 3.64), which was significantly higher than that of the control group (21.98 kg/m^2^, SD 2.90). Furthermore, fetuses born to mothers with PCOS had a higher likelihood of being SGA, and mothers in the PCOS group also exhibited an elevated risk of developing GDM.

**Table 1:** Baseline characteristics of the study population stratified by PCOS status.

Characteristic	Control	PCOS	*p* value
	*n* = 11,825	*n* = 364	
Age, Mean (SD), years	36.21 (4.00)	35.66 (3.44)	0.003
BMIG0, Mean (SD), kg/m^2^	21.98 (2.90)	23.68 (3.64)	<0.001
Birthlength, Mean (SD), cm	49.64 (2.43)	49.34 (3.38)	0.096
Birthweight, Mean (SD),kg	3274.24 (478.47)	3268.56 (486.97)	0.827
Smoking (yes)	170 (1.44%)	8 (2.20%)	0.234
Passive smoking (yes)	1,630 (13.78%)	67 (18.41%)	0.012
Drinking (yes)	758 (6.41%)	30 (8.24%)	0.162
Parity			
0	4,933 (41.72%)	248 (68.13%)	<0.001
1	6,484 (54.83%)	106 (29.12%)	
≥2	4,08 (3.45%)	10 (2.75%)	
Annual income			
Low	1,110 (9.39%)	45 (12.36%)	0.002
Middle	6,010 (50.83%)	156 (42.86%)	
High	4,705 (39.78%)	163 (44.78%)	
Normal labor (yes)	3,966 (33.54%)	136 (37.36%)	0.128
Educational level			
Primary	92(0.78%)	2(0.55%)	0.013
Junior high	624(5.28%)	12(3.3%)	
Senior high	1,280(10.82%)	24(6.59%)	
Bachelor’s degree	7,720 (65.29%)	247(67.86%)	
Master’s degree or above	2,109 (17.84%)	79(21.7%)	
GDM (yes)	3,125 (26.43%)	162 (44.51%)	<0.001
SGA	464 (3.92%)	22 (6.04%)	0.042
LGA	2,810 (23.76%)	84 (23.08%)	0.762
MAC	524 (4.43%)	22 (6.04%)	0.143
LBW	637 (5.39%)	21 (5.77%)	0.751
PTB	1,114 (9.42%)	38 (10.44%)	0.513
Newborn gender			
Male	6,140 (51.94%)	184 (50.55%)	0.653
Female	5,685 (48.06%)	180 (49.45%)	
ART	1,606 (13.58%)	123 (33.79%)	<0.001
Second-trimester ultrasound	9,052 (76.55%)	317 (87.09%)	0.265
Third-trimester ultrasound	9,256 (78.27%)	320 (87.91%)	0.405

Data are presented as Mean (Standard Deviation) for continuous variables and Number (%) for categorical variables.

PCOS, Polycystic Ovary Syndrome; BMI, Body Mass Index; GDM, Gestational Diabetes Mellitus; SGA, Small for Gestational Age; LGA, Large for Gestational Age; MAC, Meconium Aspiration Syndrome; LBW, Low Birth Weight; PTB, Preterm Birth; ART, Assisted Reproductive Technology. The participation rates in second-trimester (*p* = 0.265) and third-trimester (*p* = 0.405) ultrasound scans were comparable between the two groups, indicating no significant difference in prenatal follow-up.

*p*-values were derived from independent *t*-tests for continuous variables and Chi-square tests for categorical variables to compare differences between the control and PCOS groups.

**Figure 2:**
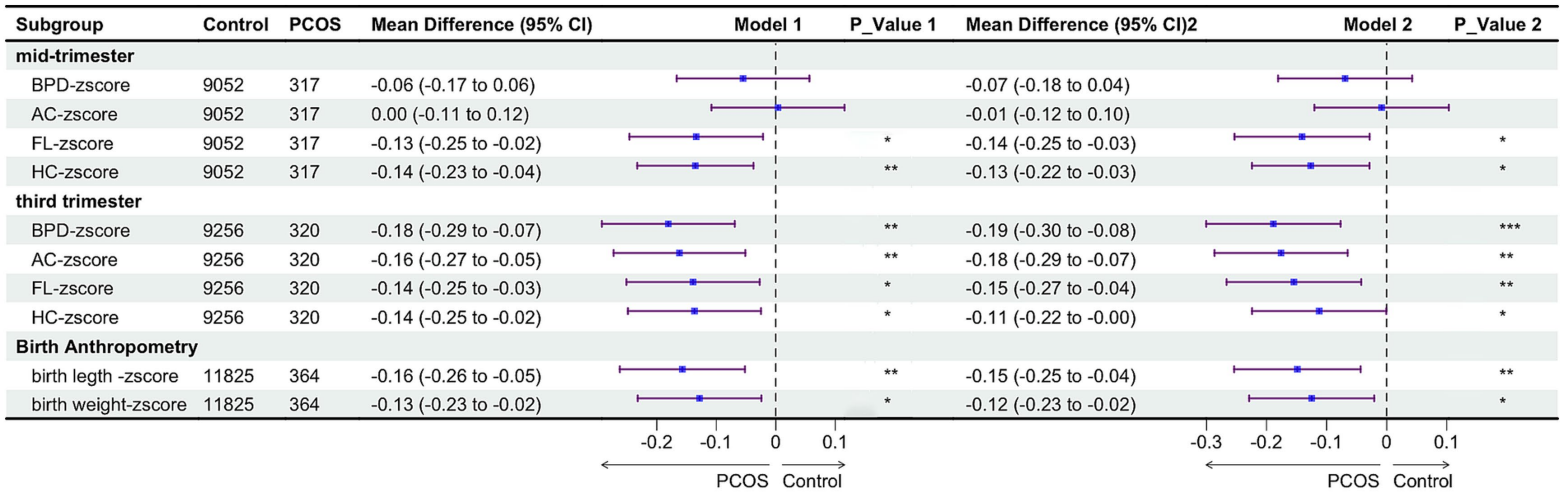
Association between PCOS and fetal growth across gestation. Women with PCOS showed a trend toward smaller fetal biometric measurements compared to controls throughout gestation. In the mid-trimester, statistically significant reductions were observed in femur length and head circumference z-scores. These differences became more pronounced in the third trimester, with significant reductions observed in all four parameters: biparietal diameter, abdominal circumference, femur length, and head circumference. Neonates in the PCOS group also exhibited significantly lower birth length and birth weight z-scores compared to controls. Data are presented as mean differences in z-scores (PCOS vs. Control) with 95% confidence intervals. Model 1 was adjusted for maternal age, pre-pregnancy BMI, smoking, drinking, passive smoking, parity, annual income, and educational level. Model 2 was additionally adjusted for gestational diabetes mellitus (GDM). BPD, biparietal diameter; AC, abdominal circumference; FL, femur length; HC, head circumference.

As shown in Figure 2, both before and after adjustment for confounders (unadjusted data are presented in Supplementary Figure 2), the z-scores for various observational parameters in the third trimester and at birth among fetuses born to women in the PCOS group were generally lower than those in the control group. Specifically, after adjusting for smoking, alcohol consumption, secondhand smoke exposure, parity, annual household income, educational level, maternal age, and pre-pregnancy BMI in Model 1, the birth length z-score (MD: −0.16; 95% CI, −0.26 to −0.05) and birth weight z-score (MD: −0.13; 95% CI, −0.23 to −0.02) in the PCOS group were significantly lower than those in the control group. The results remained significant in Model 2 after further adjustment for gestational diabetes mellitus (GDM).

Furthermore, third-trimester ultrasound findings indicated that fetuses of PCOS patients exhibited significantly lower z-scores for femur length (MD: −0.14; 95% CI, −0.25 to −0.03) and head circumference (MD: −0.14; 95% CI, −0.25 to −0.02) compared to the control population. Notably, this developmental lag was already evident in the second trimester for femur length (MD: −0.13; 95% CI, −0.25 to −0.02) and head circumference (MD: −0.14; 95% CI, −0.23 to −0.04). In contrast, a significant growth delay in biparietal diameter (MD: −0.18; 95% CI, −0.29 to −0.07) and abdominal circumference (MD: −0.16; 95% CI, −0.27 to −0.05) was not observed until the third trimester.

Among the naturally conceived population without assisted reproductive technology (ART) treatment, fetal growth in PCOS patients was significantly delayed compared to that in the control group. However, this difference was not observed in the group that conceived following ART treatment.

**Figure 3:**
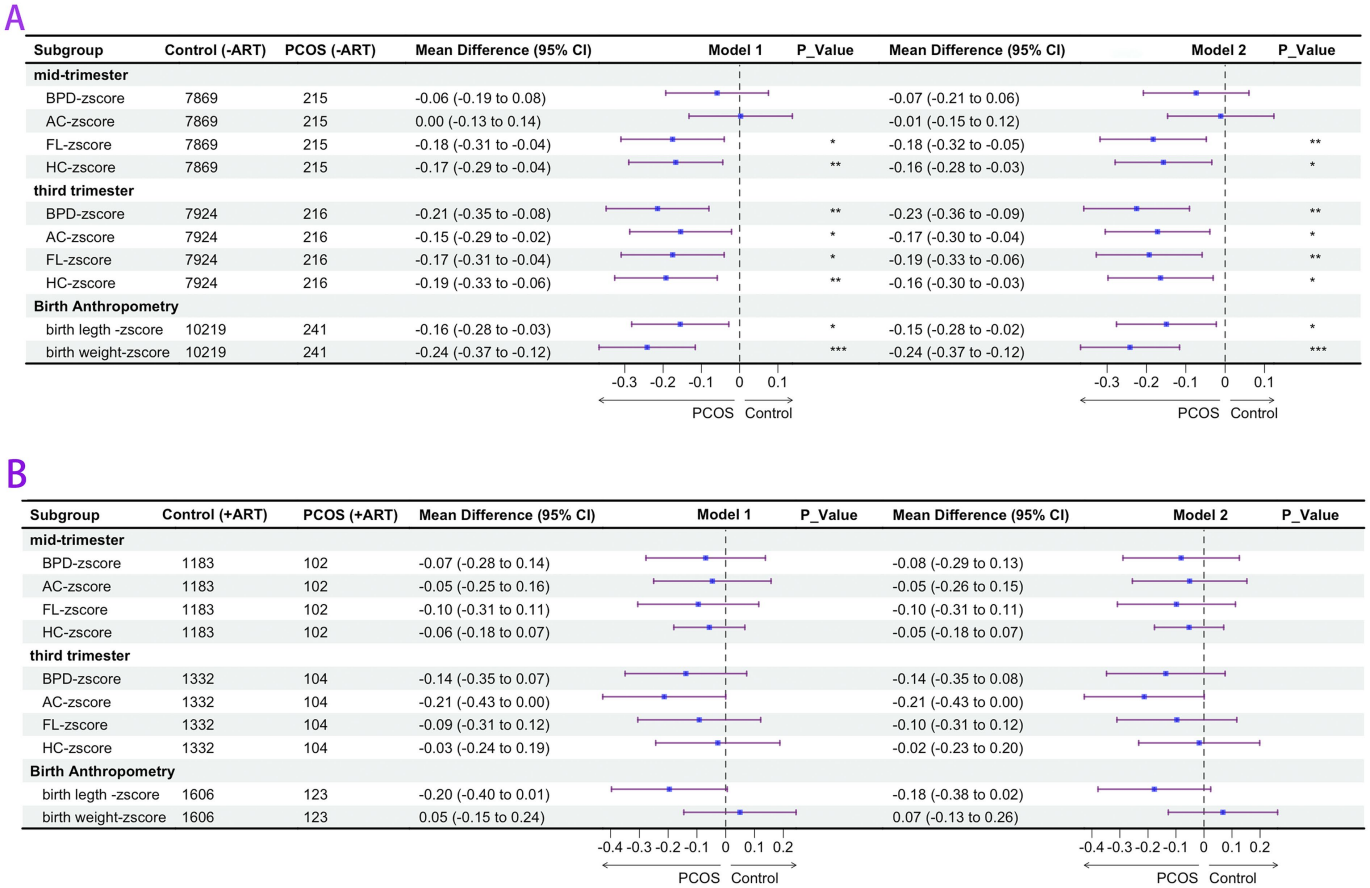
Differential associations of PCOS with fetal growth by conception modality. **(A)** Spontaneous conception subgroup. Women with PCOS showed consistently smaller fetal biometric measurements compared to controls, as indicated by negative mean differences in z-scores across most parameters in both mid- and third-trimester assessments. Statistically significant reductions were observed in femur length and head circumference during mid-trimester, and across all four parameters (biparietal diameter, abdominal circumference, femur length, and head circumference) during third trimester. Neonates in the PCOS group also exhibited significantly lower birth length and birth weight z-scores. **(B)** ART conception subgroup. No statistically significant differences in fetal growth parameters were observed between PCOS and control groups across gestation. The mean differences in z-scores for most parameters included the null value, indicating comparable fetal growth patterns between the two groups. Birth anthropometric measures showed no significant differences, with birth weight z-scores demonstrating essentially identical values between groups. Data are presented as mean differences in z-scores (PCOS vs. Control) with 95% confidence intervals. Model 1 was adjusted for maternal age, pre-pregnancy BMI, smoking, drinking, passive smoking, parity, annual income, and educational level. Model 2 was additionally adjusted for gestational diabetes mellitus (GDM). ART, assisted reproductive technology; BPD, biparietal diameter; AC, abdominal circumference; FL, femur length; HC, head circumference.

Specifically, as shown in Panel A of Figure 3, among naturally conceived PCOS patients, the z-scores for multiple fetal growth parameters were significantly lower than those in the control group. A significant decrease was already evident in the second trimester for femur length z-score (MD: −0.18; 95% CI, −0.31 to −0.04) and head circumference z-score (MD: −0.17; 95% CI, −0.29 to −0.04). This trend persisted into the third trimester, during which all observed parameters, including biparietal diameter z-score (MD: −0.21; 95% CI, −0.35 to −0.08), abdominal circumference z-score (MD: −0.15; 95% CI, −0.29 to −0.02), femur length z-score (MD: −0.17; 95% CI, −0.31 to −0.04), and head circumference z-score (MD: −0.19; 95% CI, −0.33 to −0.06), were significantly lower than in controls. Postnatal measurements followed a consistent pattern, with significantly lower birth length z-score (MD: -0.16; 95% CI, −0.28 to −0.03) and birth weight z-score (MD: −0.24; 95% CI, −0.37 to −0.12). These associations remained statistically significant in Model 2 after further adjustment for GDM.

In contrast, as shown in Panel B of Figure 3, fetal growth patterns among PCOS patients were markedly improved in the group that conceived via ART. Their growth metrics showed no significant differences compared to the control group that also underwent ART treatment. Compared to the naturally conceived population, fetal growth in PCOS patients was significantly improved among those who conceived after ART. Specifically, the data indicate that from the second and third trimesters through the neonatal period, there were no statistically significant differences in the z-scores of any growth parameters between the PCOS and control groups within the ART-treated population. These findings remained robust after adjustment for GDM.

## 4 Discussion

This study represents the first multicenter cohort research focusing on fetal development in patients with PCOS. Its strengths lie, first, in the dynamic monitoring of fetal growth parameters across the second and third trimesters, and furthermore, in the comparative analysis of the impact of ART on offspring growth in PCOS pregnancies.

Our findings indicate that fetuses and newborns of PCOS patients are associated with lower birth weight and shorter birth length. Notably, this delayed fetal growth begins as early as the second trimester and persists into the third trimester. However, among pregnancies conceived following ART treatment, the observed growth parameters approached the levels seen in the control group, suggesting that ART may, to a certain extent, mitigate the fetal growth restriction associated with PCOS.

We found that fetal growth is delayed in pregnant women with polycystic ovary syndrome (PCOS) compared to the general population, which is in line with previous studies on birth weight and length (12, 21). Furthermore, this study specifically addresses the gap in research on fetal development during the second trimester. However, prior research lacked focus on the detailed process of fetal development in the context of PCOS. Our study addresses this gap by providing evidence that fetal growth delay in PCOS pregnancies begins as early as the second trimester and exhibits a persistent pattern of delay into the third trimester. We hypothesize that fetal growth restriction in PCOS patients may be closely linked to impaired placental development. Research by Koster et al. (22) revealed significant histopathological differences in placental tissue from PCOS patients compared to controls, characterized by widespread inflammatory responses, abnormal trophoblast cell proliferation, and impaired invasiveness. This finding was further supported by a study from Palomba et al. (23), which reported that even PCOS patients without pregnancy complications exhibited significantly lower placental weight, thickness, density, and volume, indicating an inherent predisposition to suboptimal placental development in PCOS. Moreover, direct experimental evidence is provided by a recent animal model study. This research, using a mouse model of hyperandrogen-induced PCOS, found significantly impaired differentiation of trophoblast cell lineages within the placenta, ultimately leading to miscarriage in female mice (24). This result further corroborates the pivotal role of placental dysfunction in adverse pregnancy outcomes associated with PCOS.

In our cohort, various anthropometric measurements in the offspring of PCOS mothers were lower than those in the reference population. Specifically, a significant developmental lag in femur length and head circumference was observed as early as the second trimester. As gestation progressed into the third trimester, the disparities in biparietal diameter and abdominal circumference became more pronounced, with significant delays also noted in these parameters, alongside femur length and head circumference. Organs such as the skeleton and brain, which undergo rapid early development, may be particularly vulnerable to the intrauterine environment in PCOS, characterized by hyperandrogenism and insulin resistance (25, 26). This finding aligns with and may provide a fetal-period biological basis for the widely reported adverse neurodevelopmental effects observed in the offspring of individuals with PCOS, highlighting a potential link between early growth trajectories and long-term neurodevelopmental outcomes.

The design of our stratified analysis based on conception mode was informed by inconsistent reports in the literature regarding the impact of ART on PCOS pregnancies. While some studies suggest that the incidence of intrauterine growth restriction (IUGR) in PCOS patients after ART is comparable to that in the general population (27), others indicate that PCOS mothers undergoing frozen embryo transfer have a lower risk of delivering low-birth-weight or SGA infants compared to non-PCOS mothers (28). To better delineate the contribution of PCOS pathophysiology from that of ART intervention, we separately compared anthropometric measures between PCOS and reference groups within both the naturally conceived and ART-treated subgroups. Notably, we observed for the first time that growth restriction in offspring of PCOS patients was significantly ameliorated in the ART-treated group.

Previous research has shown that ART procedures may be associated with fetal growth restriction in early to mid-pregnancy, followed by compensatory placental enlargement and accelerated fetal growth toward term (29). Building on this, we speculate that growth impairment in PCOS offspring may begin earlier than observed in our study—potentially in the first trimester—due to underlying placental dysfunction (30, 31).

PCOS-related metabolic and endocrine disturbances, such as elevated androgens (32) and increased anti-Müllerian hormone (AMH) levels (33, 34), may directly or indirectly impair trophoblast invasion and angiogenesis. Excess androgens suppress decidualization of endometrial stromal cells via the AMPK/SIRT1/PDK4 signaling pathway (32), while high AMH inhibits placental aromatase Cyp19a1 expression (35), hindering the conversion of androgens to estrogens and thereby exacerbating the hyperandrogenic milieu at the maternal-fetal interface. Additionally, luteal phase deficiency and reduced progesterone levels in PCOS (36) may further compromise early placental development, which initially relies on ovarian steroidogenesis. The routine supplementation of estrogen and progesterone during ART cycles may help correct this hormonal imbalance, mitigate hyperandrogenism, and thus improve placental development, ultimately alleviating growth restriction in offspring of advanced-age PCOS patients. The timing of this intervention—early in pregnancy—appears critical, as evidenced by the absence of significant growth lag in late gestation among ART-treated PCOS pregnancies.

Moreover, PCOS is often associated with overactivation of the renin-angiotensin-aldosterone system (37) and a chronic low-grade inflammatory state (38), which may adversely affect fetal growth. The use of anti-inflammatory and immunomodulatory agents during ART could attenuate such inflammatory responses, thereby creating a more favorable intrauterine environment.

The prevalence of PCOS in our advanced-age cohort was lower than that reported in the general reproductive-age population. This may be attributed to age-related physiological changes: ovulatory dysfunction in PCOS often improves with age, and declining ovarian reserve can lead to more regular menstrual cycles, thereby rendering some patients no longer fully meeting the diagnostic criteria. Furthermore, our study included only PCOS patients with confirmed diagnoses who achieved pregnancy; cases with irregular menses but without comprehensive imaging or biochemical hyperandrogenism screening were excluded, which may also contribute to the lower observed prevalence compared to community-based estimates.

Clinical implications and future directions Understanding how conception mode influences fetal development across gestation in PCOS patients can inform individualized antenatal care strategies, enhance pregnancy monitoring, and help reduce adverse outcomes. However, translating these findings into clinical practice requires further validation in broader populations. Future research should focus on: (1) elucidating the specific molecular pathways through which hormonal supplementation and anti-inflammatory interventions during ART improve placental function in PCOS, and (2) conducting large, multicenter prospective studies to clarify the long-term effects of different PCOS phenotypes and ART protocols on offspring development, with the ultimate goal of optimizing pregnancy management in PCOS.

### 4.1 Strengths and limitations

The strengths of this study lie in its novel, multicenter cohort design, which enabled a systematic comparison of fetal growth trajectories between naturally conceived pregnancies and those in PCOS patients. It dynamically assessed the influence of assisted reproductive technology (ART) on fetal development while controlling for key confounding factors such as maternal BMI, parity, and age, thereby enhancing the internal validity of the findings.

However, several limitations should be acknowledged. First, although all PCOS diagnoses were based on established consensus criteria, the study did not perform further stratification according to phenotypic subtypes (e.g., the presence of obesity, insulin resistance, or severity of hyperandrogenism). Different phenotypes may be associated with distinct fetal growth risk trajectories; future studies incorporating such detailed stratification could enable more precise risk assessment. Second, ART was considered as a unified category without differentiating between specific treatment protocols (e.g., fresh versus frozen embryo transfer, or different stimulation regimens). This heterogeneity might obscure the effects of individual ART approaches, underscoring the need for more granular analyses in future research. Furthermore, this study was based on a cohort of women of advanced maternal age, which may have restricted the sample size and representativeness of PCOS cases. Although we adjusted for multiple confounders in the statistical analyses, prospective studies that enroll pregnant women across all age groups are still warranted to further validate the generalizability of our findings.

Despite these limitations, our core findings—that fetal growth delay in PCOS pregnancies is already evident by the second trimester and persists into the third trimester, and that this restriction is substantially ameliorated with ART treatment, bringing growth parameters to levels comparable with those of the control group—carry important clinical implications. They provide a critical foundation for early monitoring and intervention in PCOS pregnancies.

## 5 Conclusion

Our cohort study provides the first evidence that in patients with PCOS, the pathological process of fetal growth delay is already discernible in the second trimester and continues to exert an impact into the third trimester, resulting in lower birth length and birth weight compared with the reference population. Further analysis revealed that growth restriction was markedly ameliorated following ART treatment, with the related parameters largely reaching levels comparable to those of the reference population. This suggests that ART may, to some extent, mitigate growth restriction in the offspring of PCOS patients. The impact of ART treatment on the offspring of individuals with PCOS warrants further investigation.

## Data Availability

The raw data supporting the conclusions of this article will be made available by the authors, without undue reservation.
