# Supplementary material for: Fetal growth in PCOS pregnancies: dynamic evidence of restriction and the modifying role of ART
Source: Front Med (Lausanne). 2026 Jul 15;13:1857905. doi: 10.3389/fmed.2026.1857905 (PMC13415924; doi:10.3389/fmed.2026.1857905)
Supplement: Supplementary file 1 [file Supplementary_file_1.docx]

Supplementary Material

## Supplementary Figures

**
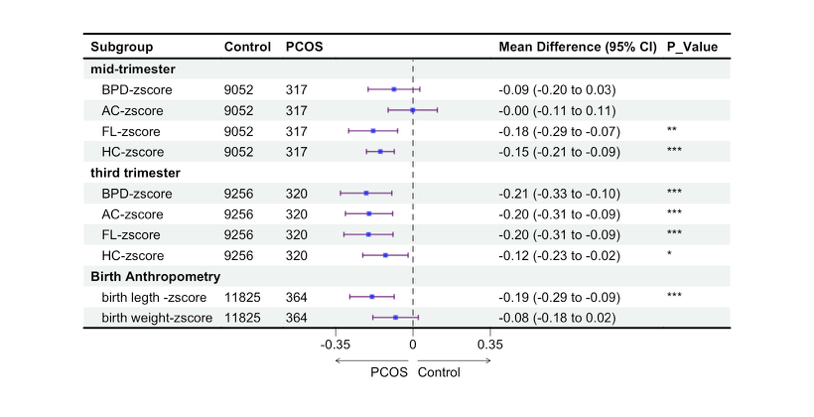
**

**Supplementary Figure 2** Unadjusted comparison of fetal growth parameters and birth anthropometry between PCOS and control groups.


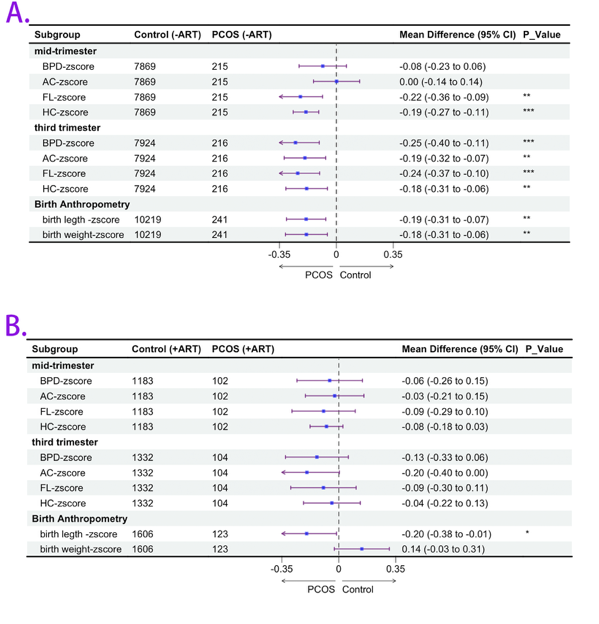


**Supplementary Figure 3** Unadjusted subgroup comparison of fetal growth parameters and birth anthropometry between PCOS and control groups, stratified by mode of conception.
